# Furnace Heat

**Published:** 1870-02

**Authors:** 


### FURNACE HEAT.

Recent discoveries announce that there is coal enough in Wyoming Territory, where every lady has recently been converted into a free-man, and can vote like every other human, to supply the world with coal for five thousand years after the millennium is over. Then, if there is plenty of coal for everybody, the best plan is to use it freely, and keep ourselves warm and comfortable and healthy, by maintaining a big fire, and letting the door stand wide open. This is the only way yet known to man of supplying families with a healthful atmosphere in severe weather.

A great deal has been written about carbonic acid, and carbonic oxide, how deathful a thing it is to breathe them, and how one turns to the other, and both of them hustle us into the grave a score or two of years before our time.

Another man comes round and tells us that unless a pan of water is kept open in a stoveroom, or near the furnace, the air will get so dry that the lungs won't work, the head will get too big for the skin which encircles it, and the breath will get hot, and finally, that the whole man will get so fidgety that the house can't contain him, and he gets his hat, tears out of the door, goes down town, and talks politics, while the poor enduring wife and the blessed little children have to stay at home and "take it," for better, for worse, day in and day out, and finally to pine away, like a flower without water, so that, between acids and oxides, and heat and cold, and hydrometric condition of the respired atmosphere, and other big names, too numerous to mention, the mass of people become so befogged that it is too troublesome to think about it, or to do any thing, and, in a kind of despair, they practically determine to let things take their course, and abide their chances, with the consolatory reflection they are no worse off than other people, who seem, by the way, to reach eighty, ninety, and a hundred years, without having ever heard o carbonic acid gas, and, consequently, never bothered themselves about it.

People lived a great while when there were no stoves nor furnaces, and they reveled in the great, old-fashioned fire-place, broad and cheery, making everybody sit around ten feet away. As there is plenty of fuel, let us go back to first principles, use Dickson's low-down grates, and have large, open fire-places, lined with brick or wood, and, as hard or soft coal, or coke, or peat, or wood can be burned in them, there is no necessary reason why they should not come into more general use.

It is very clear that iron, when made red hot, does render the air unhealthful; then, have all our stoves and furnaces lined with fire-brick, or make all stoves and furnaces out of porcelain. This is done in Germany and Russia and other nations on the other side of the Atlantic. A porcelain stove certainly gives a very genial heat, soft and delightful, in Germany, where they burn wood; but, no doubt, Yankee ingenuity could contrive one which would burn coal in New York. We used one, a year ago, in Germany, which kept parlor, dining-room, and chamber delightfully heated with wood, at a small cost, and surely wood in this country ought to cost less than in Germany, where they have to grow it for fuel, while here we burn millions of cords every year to get it out of the way.

In Russian cities the owner of a house engages to keep it warm for his tenant. The fire never goes out during the winter. Every apartment is heated by pipes bringing the heat from a distance. Why might not Philadelphia be warmed by heat made in Pottsville, or New York from a huge furnace kept in heat at Scranton, with underground pipes, which never could get red hot, surrounded with two or three feet of peat or other non-conducting material, the furnace being made of porcelain? Is there not some Field, or Lesseps, or Durant to engineer such a project through? Such a man would be a greater public benefactor than the large-hearted Peabody. Meanwhile, let us have open fire-places, and open doors, dispensing with weather-strips and double windows and all that nonsense.

